# Missing Teeth in Their Relation to Orthodontia and Bridge-Work

**Published:** 1906-09-15

**Authors:** Milton T. Watson


### MISSING TEETH IN THEIR RELATION TO ORTHODONTIA AND BRIDGE-WORK

---

BY MILTON T. WATSON, D.D.S.

---

Read before the Detroit Dental Society, March 8, 1906.

---

I shall call you attention, briefly, to the unfortunate conditions following the early loss of permanent teeth, and to the advantages and disadvantages encountered in their treatment.

The most conspicuous evil that results from missing teeth is, of course, the effect upon the occlusion. In this phase we find every thing from the simple spaces to the most pronounced irregularity, and associated with these conditions we have, often, disuse of the teeth on that particular side of the mouth where extraction has taken place and with this lack of use there is usually a diseased condition of the gums. There is also an inferior masticating capacity and the digestive disturbances that follow it.

Another exceedingly important matter to consider in relation to these cases which has never received sufficient attention, (in fact it is only within a comparatively recent period of time that it has received any consideration whatever,) is the diminished size of the maxilla where teeth are lost through early extractions, and where they are missing through failure to develop. Where permanent teeth are extracted in early childhood, the most pronounced mal-occlusion usually results, often sufficient to cause distorted and unsightly facial lines. Extractions after fifteen or sixteen years of age probably do not affect the development of the maxilla itself, and if at all, only in the slightest degree.

By a study of cases of mal-occlusion resulting from premature extractions, we will usually find a diminished nasal development and capacity; and where the extraction is confined to one side of the mouth, the teeth on the other side all being present and well arranged, the evidence is so conclusive as to convince the most skeptical.

Should you talk with a rhinologist he would tell you that many times the little patients upon whom he has operated for post nasal obstruction have not responded to the treatment as he would like to have them, and this is particularly true where the obstruction has been present for several years. It is becoming a recognized fact that children who have had a pronounced nasal obstruction for any considerable period of time, show such a lack of development in their nasal passages that the removal of the pathologic tissues alone does not restore a normal breathing space and rhinologists, as a matter of necessity, send their patients to the dentist to have the jaw enlarged to its normal size; and, by which means, they actually increase the nasal capacity. As soon as the nasal capacity is increased sufficiently so that the child can breathe entirely through the nose, then there is a development of these organs which is in harmony with the general development of the child.

Adenoid tissue usually disappears, of its own accord, between twelve and sixteen years of age, but it leaves behind it a thickened tissue that is lifeless and functionless compared with the normal tissue and this, too, in nasal passages that have never developed to a normal capacity.

It is impossible for the individual thus afflicted to receive a sufficient amount of air through the nose, and, strange as it may seem, it is said by men who have given the subject the greatest amount of thought that the mouth-breather never gets sufficient air. Mouth-breathers do not breathe as deeply and completely as normal breathers; consequently, the oxygen in their blood is not equal to that of the normal breather. You have probably noticed that these anaemic individuals have, also, a lack of nervous equilibrium.

I have, perhaps, digressed somewhat from the subject but I hope by so doing I have shown you how essential a well-arranged dental arch with the full compliment of teeth as if we expect our patients to receive the benefits accruing from normal respiration. It will, of course, be clear to you that we should avoid diminishing the size of the dental arch by extracting teeth, and that we must weigh these conditions in considering the advisability of treatment where extractions have already taken place. Dentists are thinking about teeth constantly, and most of us are making the serious mistake of thinking so much about them, alone, that we are not looking deeply enough into other conditions that are vitally associated.

Let us now consider some of the difficulties encountered in the treatment of mal-occlusion resulting from missing teeth. If we consider opening spaces, thus restoring the arch to a normal size, we are at once confronted by the necessity for mutilating sound teeth in order to put in the bridge work for restoration and retention, and the probable premature loss of the natural teeth used as abutments. I am not able to venture an opinion as to how much sooner such teeth will be lost, but it seems to be a generally accepted opinion, however, that a tooth which is compelled to do the work of two teeth, will not continue to do so throughout as long a period of time as it would, should it have only the individual work that it was originally intended to do. With adults the conditions are a little different. The difficulty is transferred from the dentist to the orthodontist, for, in these cases, we often experience great difficulty in bringing about the desired change in the position of the teeth; and oftentimes, we have experiences which tend to discourage us from doing as much of this work as we are asked to do.

The natural reluctance of adult patients to submit to orthodontic operations because of the unsightly appearance of the appliances is a matter that must be taken into consideration and the long period of retention which must be undergone is discouraging to such patients.

Where we undertake such work with older people it is well to paint them rather a gloomy picture; for, unless they start out with a clear understanding of what is before them, such patients are liable to become the very bane of a man's existence.

In addition to these features we must consider the various methods of restoring the lost teeth. It must be done securely, and inconspicuously, and in such a way that it will be easy and practicable for the patient to keep the mouth in a cleanly condition. Teeth which are inserted to hold spaces, almost invariably have a double attachment. This is a point wherein some will differ with me, but you must remember that teeth which are moved a considerable distance after the development of the alveolar process is completed are in an entirely different class from those which have become firmly and naturally attached in a normal position, and the tendency of such teeth to return to their mal-position is pronounced and continuous for a very long time. It is safer, therefore, especially in the lower jaws, to use a double attachment; otherwise, the tooth anterior to the space is liable to be crowded inside or outside the line of the arch by the backward pressure of the teeth in front of the space and every tooth anterior to it will be displaced, even around to the bicuspid region on the other side of the mouth.

Among the teeth most difficult to replace and retain, are the upper and lower anterior teeth. An upper lateral can be carried on the cuspid if the bridge work is delayed until the teeth are firmly fixed in their new position. The reason it is safer to swing this tooth from a single attachment than it is a lower, is that they have not the influence of several teeth trying to displace them as would be the case in the lower where both arches have been enlarged. It is probably true that a much longer period of usefulness would result if we should always use two attachments and I believe that molar and bicuspid replacements will only rarely, if ever, be a success when suspended from a single attachment. Diminutive teeth are no longer a source of worry to us for they can be fully replaced with an all-porcelain enamel jacket. It should be borne in mind that it is not always necessary, or even desirable with adult patients, to open spaces where teeth have been lost, for we can often close a space by carrying all teeth posterior to the space, forward of their natural occlusion, one cusp.

I have recently seen some of the removable bridge work done by Dr. Peeso, of Philadelphia. It is the most beautiful gold work that I have ever seen and the fact that the attachment is not so rigid as that of the shell crown, is to my mind a great advantage for it permits a slight movement which is normal in all teeth. I believe the longevity of teeth used for abutments, in this class of bridge work should be much greater than where the attachment of the bridge is cemented securely.

DISCUSSION.

Dr. White: My own boy, when three years old, started to develop a nasal obstruction. He had, at the time, a perfect occlusion, and in five months more he had developed decided protrusion of the upper centrals, so that you can readily see how such a nasal obstruction will affect a child in such a short time. I had him operated upon about four weeks ago. Of course, I cannot see any change in him in so short a time. I do not think the arch will be affected in the future. This is something that it is very hard for the general practitioner to see, as we do not get the children in at so young an age. We see them often at the age of four, five or six, and we should be very careful to notice anything of that kind and we can do more good in calling the parents attention to it. I know in the case of my boy that it would, without a doubt, have been a very disastrous case if we had not interferred.

Dr. Hoff: I do not know whether Dr. Watson brought out the matter clearly as to whether these nasal obstructions are the prime causes of contracted arches, or whether they are caused by the contracted arches. From what Dr. Watson says, and most of our rhinologists say, I presume we would be justified in concluding that it is the nasal obstruction from adenoid growths, or a lack of development of the facial bones, which, in a large measure, accounts for the crowded condition of the arch, and perhaps, for the malposition of the teeth, as much as absent and faultily developed teeth. It seems to me that we have not yet enough data on these points to enable us to always arrive at a correct diagnosis of these cases, or to make definite statements about them. My own opinion is that nasal obstructions from pathologic conditions are more often the probable cause than any neglect of treatment of mal-occlusion of the dental organs. There are cases, no doubt, where the dental organs are at fault, as when mal-occlusion is associated with an evident lack of dental service at the proper time. For instance, in the too early extraction of the deciduous teeth, or the too long retention of them.

There is a class of cases of missing teeth that it has always puzzled me to account for, that I should like someone to give me some information as to their treatment. These are the conditions where we have missing teeth in arches that are, so far as we can determine, of the proper size, and no extractions have occurred, the teeth more commonly missing are the lateral incisors. Frequently we see cases where one or both of the upper lateral incisors are missing and there is a wide space between the centrals. A space is also left where the lateral incissors should be, but no indication of these teeth in the jaw anywhere, and a radiograph reveals no sign of them. In observing these cases you will often note that the cuspid teeth are not long, nor do they have well-developed crowns; always short crowned, or, as it seems, they do not extrude from the gum as far as the ordinary tooth does, and the central incisors are usually not perfectly formed teeth.

The mesial and distal surfaces are square or angular rather than having that round or symmetrical form which these teeth ought to have.

With reference to treating these cases, I have in mind two patients in my own practice. The spaces between the central and cuspid teeth are not so large and broad as to make a positive deformity, and yet everyone who sees these patients knows that there is something wrong with their teeth.

One patient, a little girl, whose father has never erupted one lateral, and her mother also has never erupted them. In the father's case the teeth are close together so that there is no deformity; in the mother's case there are rather wide spaces between the teeth. I have wondered how I should treat this child. Whether to separate them and insert a lateral incisor on each side by bridging, and if so, what would be the best way to bridge so that it would be firm and inconspicuous, unless I mutilate either the cuspid or the centrals.

In these cases of lack of development it is a very different proposition than where they are lost from disease.

Dr. Thompson: I would like to ask Dr. Watson, in the operation for removal of adenoids, how much space is really gained. I heard a physician say yesterday that a great many operators admitted that there was a contraction of the tissue after the operatiou and that really it left very little more space than there was in the first place. It was a new idea, I supposed, where a great deal of tissue has been removed, we should have considerable scar tissue and subsequent contraction.

Dr. Spalding: Dr. Watson says pyorrhea is aggravated by mal-occlusion. I do not remember that I have ever seen a case of pyorrhea in a mouth with normal occlusion, and I think that there is almost no irritation of the gum tissue because nature has given us an apparatus which is really self-cleansing, as Dr. Black puts it, the natural run of the food will keep the surface clean. When a few teeth are lost the other teeth soon drift into bad occlusion, and every protected surface will serve to accumulate ptomaines, and where these accumulate there will nearly always be inflammation. Among the lower teeth, with the first molars missing, it is seldom that the second molar does not tilt forward, then the mesial surface accumulates mucoid deposits which decompose and infect the gum and produce inflammation and finally pyorrhea. Nearly every variation from the normal occlusion makes a condition which invites inflammation of the gum tissues or caries.

We cannot deny that it is necessary, in a great many cases to supply missing teeth, but the ideal bridge work certainly has not been suggested or brought out yet. I think any useful and substantial bridge work which can be removed by the patient enables the mouth to be kept in a more cleanly condition, and the abutments also can be more easily cleaned. Dr. Peeso, of Philadelphia, opens a good field for prophylaxis results. He has some beautiful work, but there will be the objection, to my mind in children, that often he requires the devitalization of teeth to insert the tube, and in young children where the teeth are not fully developed, it might be an objection. He probably has some other attachments for such cases, which I do not know of.

Dr. Wood: I may be wrong, but I have, to my own satisfaction, treated two or three of these cases and devitalized an adjoining tooth and set posts with backing into the two teeth to supply one. I had a very solid case when I got through and I felt justified in doing that.

Dr. Hoff: The important point of the case I mentioned is that the child is a beautiful girl of eight or nine years. There is no indication of any lateral incisors coming in her mouth. In fact, the skiagram shows no teeth in the jaw. If this child should have those lateral incisors lost, as her mother and father both have, it is going to spoil her beauty. I do not want to devitalize the little girl's cuspid teeth in order to cut them off and put crowns on them.

Dr. Bowles: This paper has two lessons; one is to be very careful about extracting teeth. We have heard a great deal about how these cases can be corrected. The necessity for the correction should not be made. The second lesson is to let these cases alone. In times past I have undertaken, occasionally, to regulate cases of mal-occlusion. When I hear of the distress of a specialist like Dr. Watson, I have concluded that the thing for me to do is to refer all such cases to him.

One point he made is that mouth-breathers do not breathe so freely as those who breathe through their nasal passages. It seems to me that I can breathe more freely with open mouth than with it closed, as will be illustrated by the fact that when running it is natural for one to open the mouth and breathe through it.

A question has been asked, suppose we have a case with the cuspid just protruding through the alveolar process and the first bicuspid occupying the space that the cuspid should occupy, after all the difficulties that Dr. Watson has enumerated in regard to moving those teeth back, whether the best thing would not be simply to extract that first bicuspid and let the cuspid come down. I merely mention this so that Dr. Waston can dilate on that subject when it comes his turn again.

Dr. Watson: Dr. Thompson asked about the possible increase of the nasal breathing capacity in children who have adenoids removed surgically. That depends entirely on the nature of the case. We sometimes find adenoid tissue hanging from a comparatively small space, the removal of which would not leave scar tissue of much importance. We find other cases where the tissue spreads out almost covering the eustachian tubes, and the removal of such a band of tissue is liable to be followed by considerable contraction and scar tissue, yet the fact remains that the children who have large masses removed, who were unable to breathe through their noses before the operation, can breathe normally afterwards.

Operators entertain different ideas as to how these operations should be performed, some resorting to most heroic methods, which, of course, leaves a much greater scar area. Adenoid cases require careful watching for a second operation is often necessary. They often recur in cases where the adenoids remained for a considerable period of time before removal and where the nasal passages as a whole are undeveloped.

Dr. Hoff asks the question whether the nasal obstructions cause mal-occlusion or mal-occlusion causes nasal obstructions. You must remember that in all cases of mal-occlusion of the teeth it is not always adenoid tissue that is at fault. When we stop to consider that frequently pronounced nasal occlusion is established at one year of age, it is not difficult to decide which precedes the other, when the two are associated.

Dr. Bowles spoke of opening his mouth when running in order to get more air. You must remember that under such circumstances a man is also breathing more or less through his nose at the same time. A man who has a restricted nasal passage would have a greater capacity through his mouth. I know that the most eminent authorities are of one mind that the mouth breather never breathes so deeply and completely as the normal breather and I intended to convey just that impression and no other.

The case Dr. Bowles refers to in which a cuspid is just erupting, and where the bi-cuspid has moved forward, would not, of necessity, be difficult to treat, though such a case without other complications would be a rare one indeed.

